# Predictors of Burnout in Hospital Health Workers during the COVID-19 Outbreak in South Korea

**DOI:** 10.3390/ijerph182111720

**Published:** 2021-11-08

**Authors:** Chang-Ho Jihn, Bokyoung Kim, Kue Sook Kim

**Affiliations:** 1 Department of Industrial and Management Systems Engineering, Kyung Hee University, Yongin 17104, Korea; jihn@khu.ac.kr; 2 Department of Nursing, Catholic Kwandong University, Gangneung 25601, Korea; ilt10@hanmail.net; 3 Health Care Center, Seoul Metropolitan Dong Bu Hospital, Seoul 02584, Korea

**Keywords:** burnout, COVID-19, hospital health worker, doctor, nurse, emotional exhaustion, depersonalization, personal accomplishment, Maslach burnout inventory

## Abstract

This study aimed to identify the factors that influence the components of burnout—emotional exhaustion (EE), depersonalization (DP), and personal accomplishment (PA)—among hospital health workers, including doctors and nurses, during the COVID-19 pandemic. We analyzed 200 healthcare workers’ responses to the Employee Health Promotion Survey conducted at a general hospital in Seoul with over 200 hospital beds. The questionnaire included items about COVID-19-related burnout and its influencing factors. We performed three different multiple regression analyses using EE, DP, and PA as the dependent variables. The results show that sex, marital status, workload of treating suspected COVID-19 patients, fear of COVID-19 infection, anxiety, and depression predicted EE. The predictors of DP were job category, consecutive months of work in the current department, satisfaction with work environment, anxiety, and depression. The predictors of PA were the workload of directly interacting with patients, socioeconomic status, and job stress. For EE and DP, burnout was found to be worse in doctors and nurses than in other health workers; moreover, burnout was worse among nurses than among doctors across all three aspects of burnout. The findings can be used to establish tailored policies to address each burnout component.

## 1. Introduction

According to a report by the World Health Organization (WHO), as of 15 September 2021, there were approximately 230 million confirmed cases of COVID-19 worldwide. In South Korea, 277,989 confirmed cases and 2380 deaths have been reported [1]. The WHO defines health workers (HWs) as “all people engaged in actions whose primary intent is to enhance health” [2]. These HWs constitute the core workforce when encountering infectious diseases such as COVID-19. As a result of their role in managing and maintaining medical services at the front line during the spread of infectious diseases, HWs—such as doctors, nurses, midwives, paramedical staff, hospital administrators, support staff, and community workers—face a higher risk of infection than the general public. Furthermore, these workers are exposed to risks such as psychological distress, fatigue, and stigma [3]. According to a report from the International Council of Nurses (ICN), as of February 2021 [4], the average infection rate across the ICN dataset ranges between 6% and 10% at different points in time and HW infection rates of up to 30% have been reported. In South Korea, 565 health practitioners tested positive for COVID-19 while treating patients between February 2020 and June 2021. Of these, 20.0% were doctors and 73.5% were nurses, with the higher number of the latter likely due to the distinctive nature of nursing tasks in the field of disease prevention and patient care [5]. High infection and death rates among HWs can affect the maintenance of the healthcare system.

Amid the prolonged COVID-19 pandemic, HWs are complaining about accumulated fatigue and mental stress, and are suffering from burnout due to constant labor shortages and insufficient benefits [6]. Burnout refers to “a psychological syndrome of emotional exhaustion (EE), depersonalization (DP), and reduced personal accomplishment (PA) that can occur among individuals who work with other people in some capacity” [7]. EE is the depletion of emotional resources, DP is developing a cynical attitude toward patients, and reduced PA is a negative evaluation of oneself [7]. The head of the Korean Health and Medical Workers’ Union reported that HWs frequently quit their jobs because of extreme fatigue and exhaustion, and that many workers suffer from extreme physical and mental stress, which leads to depression and trauma. Moreover, HWs experiencing burnout can impact patients and colleagues as they have an increased risk of making wrong decisions [8]; burnout is therefore not an issue that only pertains to individual HWs. However, sufficient measures have not been taken to resolve the poor working conditions and heavy workloads for HWs [9].

Previous studies that analyzed burnout after the outbreak of COVID-19 limited their research subjects to single job categories, such as doctors [10,11,12] and nurses [13,14,15]. Such fragmentary analysis of burnout, with a focus on a particular job category, may be helpful for making policy decisions pertaining to a particular job category. However, these studies overlooked the actual clinical setting where multiple job categories are organically interconnected within one hospital and that burnout during a pandemic, such as COVID-19, affects the entire hospital. Although one study investigated the burnout of HWs from multiple hospitals [16], it was difficult for the researchers to build three individual models for the levels of EE, DP, and PA under the same conditions in one hospital and to identify relevant influencing factors.

For this reason, we aimed to differentiate our study from previous studies by including all HWs working at the same hospital. Departing from the previous pattern of analyzing job-oriented burnout, we employed an exhaustive analysis method focusing on the organization. This analytical approach can assist hospital personnel in charge of healthcare policy to gain a comprehensive understanding of the challenges confronting hospital HWs and to make efficient decisions. In addition, by implementing an employee burnout prevention policy that is applicable to all job categories, hospital HWs can perform their jobs more cost effectively. Thus, we aimed to provide a foundation and baseline data for establishing hospital-level policies on burnout prevention right at the early stages when encountering a pandemic such as COVID-19.

The overarching research question was: “What factors affect hospital HWs’ burnout during the COVID-19 outbreak in terms of the individual burnout components?” The hypotheses were:

**Hypothesis** **1** **(H1):** *Sociodemographic characteristics and variables related to COVID-19, work overload, psychological conditions, and hospital resources will affect the EE of hospital HWs during the COVID-19 outbreak.*

**Hypothesis** **2** **(H2):** *Sociodemographic characteristics and variables related to COVID-19, work overload, psychological conditions, and hospital resources will affect the DP of hospital HWs during the COVID-19 outbreak.*

**Hypothesis** **3** **(H3):** *Sociodemographic characteristics and variables related to COVID-19, work overload, psychological conditions, and hospital resources will affect the PA of hospital HWs during the COVID-19 outbreak.*

## 2. Materials and Methods

### 2.1. Study Design

This retrospective descriptive study explored the factors affecting burnout among HWs at a Korean hospital during the COVID-19 pandemic.

### 2.2. Participants and Sample Size Calculation

This study’s subjects included the participants of the 2020 Employee Health Promotion Survey for HWs working at a general hospital in Seoul equipped with over 200 hospital beds during the COVID-19 pandemic. Data were collected during the comprehensive wellness check from 6 January 2020, to 28 February 2020. The total number of employees in the hospital is 311, of which 210 responded to the questionnaire. The overall response rate was 64%. After excluding 10 people who did not submit their responses within the collection period, only 200 questionnaires were used as valid data in this study. The 200 responses that had already been collected were used for the final analysis. Using the G*Power 3.1.9.7 program [17], the minimum sample size for multiple regression analysis was calculated to be 183 based on a previous study [18] with a significance level of 0.05, a median effect size of 0.15, a power of 0.90, and a number of predictors at 18. Therefore, a sample size of 200 for this study was appropriate.

The composition of the 200 participants is as follows: 48 doctors, 83 nurses, 6 pharmacists, 28 health workers, 7 managers, 3 technical workers, and 25 service workers. The response rates by occupational group were: 100% for doctors, 52% for nursing, 100% for pharmaceutical workers, 100% for health workers, 26% for managers, 38% for technical workers, and 72% for service workers.

### 2.3. Instruments

#### 2.3.1. General Characteristics

The questionnaire on general characteristics consisted of 13 items as follows: sex, age, education level, marital status, job category, working duration at the current job, working duration in the current department, number of rotating shifts within a month, workload of directly interacting with patients, workload of treating suspected COVID-19 patients, socioeconomic status, satisfaction with work environment, and current health condition. Age details were collected as an ordinal variable. Using Likert scales, we categorized and measured the following variables: education level, workload of directly interacting with patients, workload of treating suspected COVID-19 patients, socioeconomic status, satisfaction with work environment, and current health condition. Nominal variables with *c* classes were represented by *c* − 1 dummy variables, each taking on the values 0 and 1.

#### 2.3.2. Fear of COVID-19 Infection

Fear of COVID-19 infection was measured using a revised scale based on the fear of MERS-CoV infection [18] scale. This scale consisted of one item: “I am afraid of being infected with COVID-19”, which was measured using a 10-point Likert scale, whose values ranged from 1 (not at all afraid) to 10 (unbearably afraid). A higher score indicated a stronger fear of COVID-19 infection.

#### 2.3.3. Job Satisfaction

Job satisfaction was measured using the Minnesota Satisfaction Questionnaire, which was developed by the Minnesota Industrial Relation Center [19] and translated into Korean by Lee and Park [20]. This questionnaire consisted of 20 items: 10 items about intrinsic factors and 10 about extrinsic factors. Using a 5-point Likert scale, responses were measured from 1 (very dissatisfied) to 5 (very satisfied). A higher score indicated a higher level of job satisfaction. Cronbach’s α, the reliability indicator of the scale, was 0.88 in Lee and Park’s [20] study, and 0.97 in this study.

#### 2.3.4. Hospital Anxiety and Depression

Hospital anxiety and depression levels were measured using the Hospital Anxiety and Depression Scale (HADS) developed by Zigmond and Snaith [21], and standardized by Oh et al. [22]. The HADS consists of 14 items: 7 items about anxiety and 7 items about depression. Responses were measured on a 4-point Likert scale ranging from 0 (never) to 3 (frequently). A higher score indicated a higher level of anxiety or depression. As 8 was suggested as the cut-off score in a previous study [22], we regarded scores above 8 as a manifestation of anxiety or depression. The Cronbach’s α of the scale was 0.89 for anxiety and 0.86 for depression in Oh et al.’s study [22]. In this study, it was 0.687 for anxiety and 0.76 for depression.

#### 2.3.5. Job Stress

Job stress levels were measured using the Korean Occupational Stress Scale Short Form (KOSS-SF) [23]. The KOSS-SF consists of seven subscales and 24 items as follows: job demand (4 items), insufficient job control (4 items), interpersonal conflict (3 items), job insecurity (2 items), occupational system (4 items), lack of reward (3 items), and organizational climate (4 items). Using a 4-point Likert scale, the scores were reverse-coded from 1 (strongly disagree) to 4 (strongly agree). The stress score of the KOSS-SF was calculated by converting the scores of the seven subscales into a 100-point scale and averaging them. The Cronbach’s α for each subscale at the time of KOSS’s development was as follows: 0.71 for job demand, 0.66 for insufficient job control, 0.67 for interpersonal conflict, 0.61 for job insecurity, 0.82 for occupational system, 0.76 for lack of reward, and 0.51 for organizational climate [23]. In this study, the overall reliability of the scale was 0.89, and that of each subscale was: 0.56 for job demand, 0.77 for insufficient job control, 0.77 for interpersonal conflict, 0.74 for job insecurity, 0.89 for occupational system, 0.81 for lack of reward, and 0.82 for organizational climate.

#### 2.3.6. Hospital Resources for the Treatment of COVID-19

Hospital resources for the treatment of COVID-19 were measured using a revised scale based on Kim and Choi’s scale of “Hospital Resources for the Treatment of MERS-CoV” [18]. That is, we replaced the term MERS-CoV used in their scale with COVID-19 as follows: “My hospital is equipped with facilities sufficient for preventing the spread of COVID-19”, “My hospital applies the best infection control guidelines for preventing the spread of COVID-19”, and “My hospital discusses how to prevent COVID-19 regularly”. Based on a briefing on the supply management plan for COVID-19 by the Central Disaster and Safety Countermeasures Headquarters [24], the following two items were added: “My hospital supplies facemasks steadily” and “My hospital steadily supplies personal protective equipment (gloves, bodysuit, goggles, hood, etc.)”. The scale consisted of five items, which were scored on a 4-point Likert scale ranging from 1 (strongly disagree) to 4 (strongly agree). A higher number indicated a greater availability of hospital resources for encountering COVID-19. The Cronbach’s α was 0.81 in a previous study [18], and 0.84 in this study.

#### 2.3.7. Support from Family and Friends

Support from family and friends was measured using a revised scale based on Kim and Choi’s scale of “Support from Family and Friends”, which focused on the MERS-CoV epidemic [18]. Based on their scale, our scale was revised by replacing the term MERS-CoV with COVID-19 as follows: “My friends will avoid me if they find that I have cared for COVID-19 patients”, “My friends will support me caring for COVID-19 patients”, “My family will avoid me if they find that I have cared for COVID-19 patients”, and “My family will support me caring for COVID-19 patients”. The scale consisted of four items and was measured using a 4-point Likert scale ranging from 1 (strongly disagree) to 4 (strongly agree). A higher score indicated stronger support from family and friends. The Cronbach’s α was 0.80 in a previous study [18], and 0.57 in this study.

#### 2.3.8. Working Overtime and Compensation Related to COVID-19

The scale measuring working overtime and compensation related to COVID-19 consisted of 2 items as follows: “My hospital requires me to work overtime because of COVID-19” and “My hospital pays extra for working overtime because of COVID-19”. Using a 4-point Likert scale, the responses were measured from 1 point (strongly disagree) to 4 points (strongly agree), and additional pay was reverse-coded. A higher score indicated a higher frequency of working overtime without proper compensation.

#### 2.3.9. Burnout

Burnout was measured using the Korean version [25] of the Maslach Burnout Inventory Scale Human Services Survey (MBI-HSS) developed by Maslach and Jackson [26]. The MBI-HSS differs from MBI for medical personnel (MBI-HSS (MP)) [27] in terms of the choice of words in the items: “recipients” vs. “patients”. Accordingly, we replaced the corresponding words with the Korean translations. Similarly, Jung’s [25] Korean MBI-HSS version was used in a study of HWs at community health centers [28].

Both MBI-HSS and MBI-HSS (MP) consist of three independent subscales: EE, DP, and PA. Each questionnaire comprises 22 items: 9 items about EE, 5 items about DP, and 8 items about PA. The responses were measured using a 7-point Likert scale as follows: 0 points for “Never”, 1 point for “Less than once a year”, 2 points for “Less than once a month”, 3 points for “2–3 times a month”, 4 points for “Once a week”, 5 points for “2–3 times a week”, and 6 points for “Every day”. High EE and DP with low PA scores indicated a higher level of burnout. The commonly used cut-off points of EE, DP, and PA are 27, 10, and 33, respectively [29].

Following the MBI advice that the sum of all subscale scores is not an ideal indicator of burnout [26,27], we calculated the scores per subscale and interpreted them separately. Cronbach’s α, the test score reliability indicator for each subscale at the time of scale development by Maslach and Jackson [30], was 0.90 for EE, 0.79 for DP, and 0.71 for PA. The reliability of the subscales in this study was 0.92 for EE, 0.84 for DP, and 0.90 for PA.

### 2.4. Ethical Considerations and Data Collection

Before data collection, we obtained approval from the institutional review board (IRB) of the Seoul Medical Center regarding adherence to ethical guidelines and permission to view the responses (no. SEOUL 2021-01-002-003). The collected responses did not contain any information that could identify the participants. To avoid data leakage, we viewed the data only in the office of the hospital of the employee health promotion team. Moreover, the document file was encrypted with a password and saved on a computer that could only be accessed by the researcher. A total of 200 responses were retrieved and used in the analysis. The research data file will be disposed of three years after the research is complete.

### 2.5. Data Analyses

We used R and SPSS for Windows (version 26.0; IBM Corp., Armonk, NY, USA) to analyze the data. The data set included the following variables: general characteristics of the participants, burnout related to COVID-19, fear of COVID-19 infection, job satisfaction, anxiety, depression, job stress, available hospital resources, additional compensation for overtime work, and support from family and friends. The reliability of the scales was measured using Cronbach’s α. Regarding the general characteristics of the participants, we calculated the frequency, percentage, mean, and standard deviation. To analyze the differences in burnout components (EE, DP, and PA) according to the general characteristics, we used the following tests: Student’s *t*-test, Welch’s *t*-test, analysis of variance, Kruskal–Wallis test, and Scheffe’s post hoc test. Pearson’s correlation test was used to analyze correlations. These test results were used to ensure that only informative variables were selected to avoid the curse of dimensionality. To explore the factors that affected burnout related to COVID-19, we performed three different multiple regression analyses, where the explanatory variables were the statistically significant variables from the difference tests and correlation analysis, and the dependent variables of burnout were the EE, DP, and PA scores.

## 3. Results

### 3.1. General Characteristics

Most of the participants were women, college graduates, and health practitioners. In terms of satisfaction with the work environment and health condition, they perceived it to be above average. The number of female participants was approximately three times higher than that of male participants. Regarding age, the ratio of participants under 40 and above 40 years was almost the same. Moreover, the number of married people was 1.6 times higher than that of single people. As for job type, nurses accounted for 41.5% and doctors accounted for 24.0%. The other health workers (OHWs) accounted for 34.5% of the participants. Of all participants, 73.5% belonged to a job category that required interaction with patients. Participants who were likely to interact with suspected COVID-19 patients accounted for 31.0%. Of all participants, 72.0% perceived themselves as having a middle or high socioeconomic status. Regarding satisfaction with the work environment, the ratio of satisfied and dissatisfied was approximately 6:4 (Table 1).

### 3.2. Burnout and Other Variables

The mean EE, DP, and PA scores were 26.48, 11.03, and 28.47, respectively. These were worse than the means of a medicine group (*n* = 1104) provided for comparison in the fourth edition of the MBI manual [27], where the mean EE, DP, PA scores were 22.19, 7.12, and 36.53, respectively. The median fear of COVID-19 infection was 6 and its interquartile range (IQR) was 4. Regarding job satisfaction, the mean was 50.92, which was between “satisfied” and “slightly satisfied”. Regarding anxiety and depression, the means were 7.49 and 8.87, respectively, almost or above the cut-off point (8 points). Regarding job stress, the mean was 49.09, which was close to the median. As for hospital resources for COVID-19 and support from others, the mean was close to the median and between “slightly disagree” and “slightly agree” (Table 1).

### 3.3. Difference Testing, Correlation Analysis, and Multiple Regression Analysis

Based on the mean difference test and correlation analysis of EE scores, the variables that were found to be statistically significant were as follows: sex, age, education level, marital status, job category, number of rotating shifts within a month, workload of directly interacting with patients, workload of treating suspected COVID-19 patients, satisfaction with work environment, current health condition, fear of COVID-19 infection, job satisfaction, anxiety, depression, job stress, and hospital resources for COVID-19. Based on the mean difference test and the correlation analysis of DP scores, the variables that were found to be statistically significant were as follows: sex, job category, working duration in the current department, workload of treating suspected COVID-19 patients, satisfaction with work environment, current health condition, fear of COVID-19 infection, job satisfaction, anxiety, depression, job stress, and hospital resources for COVID-19. Based on the mean difference test and the correlation analysis of PA scores, the variables that were found to be statistically significant were as follows: age, education level, doctor, workload of directly interacting with patients, socioeconomic status, job stress, and working overtime and compensation. For all the mean difference tests and correlation analyses, the significance level of 0.05 was used.

We used the Durbin–Watson test to detect the presence of autocorrelations. The heteroscedasticity and autocorrelation consistent (HAC) covariance matrix estimation were used to overcome autocorrelations [31]. The variance inflation factor was used to test for multicollinearity. All variables had variance inflation factor (VIF) values between 1.118 and 2.465, indicating no multicollinearity.

Based on the multiple regression analysis of EE, the variables that were found to be statistically significant included sex, marital status, workload of treating suspected COVID-19 patients, fear of COVID-19 infection, anxiety, and depression. Significant variables for DP included nursing, working duration in the current department, and anxiety. When adjusting the significance level up to 0.10, satisfaction with the work environment and depression were included. Significant variables for PA included workload of directly interacting with patients, socioeconomic status, and job stress. The regression models of EE, DP, and PA explained 52.08%, 34.98%, and 14.94%, respectively, of the variance in their scores for COVID-19-related burnout (Table 2).

### 3.4. Hierarchical Regression Analysis

We performed three sets of hierarchical regression analyses (HRA) to see whether adding four different groups of variables significantly improved the model’s ability to predict the burnout. For each analysis, the predictor variables were entered within four successive steps. The order in which groups of variables were added is as follows: sociodemographic group, COVID-19 group, work overload group, and psychological group. The groups for EE, DP, and PA were constituted by statistically significant variables in Table 2 for each component type. Thus, the composition of each group varies depending on the component type. A sociodemographic group can be constituted of sex, age, education level, marital status, socioeconomic status, current health condition, nursing, or doctor. A COVID-19 group can be constituted of fear of COVID-19 infection, workload of treating suspected COVID-19 patients, or hospital resources for COVID-19. A work overload group can be constituted of working duration in the current department, number of rotating shifts within a month, workload of directly interacting with patients, satisfaction with work environment, job satisfaction, or working overtime and compensation. A psychological group can be constituted by anxiety, depression, or job stress.

Regarding EE, in the first step, the sociodemographic group accounted for 28.0% of variance in burnout (*p* < 0.001). In the second step, the COVID-19 group explained an additional 9.9% of the variance (*p* < 0.001). In the third step, the work overload group explained an additional 4.8% of the variance (*p* < 0.05). In the fourth step, the psychological group explained an additional 13.4% of the variance (*p* < 0.001).

Regarding DP, the group of the first step accounted for 14.8% of variance in burnout (*p* < 0.001). The groups of the second, third, and fourth step, respectively, explained an additional 4.7% (*p* < 0.001), 6.4% (*p* < 0.05), and 12.9% (*p* < 0.001) of variance in burnout.

Regarding PA, the COVID-19 group was not considered for the HRA since there is no statistically significant variable of the COVID-19 group in Table 2. In the first step, the sociodemographic group accounted for 10.6% of variance in burnout (*p* < 0.001). The work overload group in the second step and psychological group in the third step, respectively, explained an additional 3.5% (*p* < 0.05) and 3.8% (*p* < 0.05) of the variance in burnout.

## 4. Discussion

This study is significant in that it investigated the burnout of all HWs working at the same hospital during the early stages of treating the COVID-19 pandemic, and analyzed the factors of burnout from multiple perspectives in terms of its three components (EE, DP, and PA). This section explains burnout based on the significant variables of each component and proposes concrete strategies to mitigate burnout.

### 4.1. Situation of Hospital Health Workers in South Korea Compared to Other Countries

According to a survey of possible burnout for 2707 healthcare professionals in 60 countries in April 2020, 51% of respondents reported burnout [32]. In addition, in a systematic review analyzing 11 studies of healthcare professionals’ burnout conducted mainly in April–May 2020, the prevalence of overall burnout was 49.3% to 58% [33]. In Korea, as a result of a regular survey of about 67,000 health and medical workers conducted by the Korean Health and Medical Workers’ Union (KHMWU) in February 2019, before the outbreak of COVID-19, 70.6% of respondents complained of physical and mental burnout. This is even higher compared to the reported burnout rate during COVID-19 in other countries. As a result of a regular survey conducted in March 2021 by KHMWU in about 43,000 health and medical workers, 69.6% of the respondents complained of physical exhaustion, and 65.8% of them were mentally exhausted. Of the total respondents, 78.7% answered that their daily life had deteriorated, and 70.6% of the respondents answered that their psychological state also deteriorated [34,35]. As such, Korean HWs report chronic burnout every year, and they endure daily physical exhaustion and emotional labor.

### 4.2. Burnout Level of Korean Hospital Health Workers during the COVID-19 Outbreak

The results show that the Korean participants in this study had much greater risks for burnout in all three aspects (EE, DP, and PA) than other countries. Regarding the means of each component during the COVID-19 outbreak, the mean of the participants’ EE was 26.48 points out of 54 points, DP was 11.03 points out of 30 points, and PA was 28.47 points out of 48 points. By comparison, a study conducted among frontline healthcare professionals who worked during the peak of the COVID-19 pandemic in Italy showed the following: 22.7 points in EE, 6.1 points in DP, and 37.5 points in PA [36]. In another study carried out among health professionals in Italy [37], the means were 22.3 points in EE, 4.7 points in DP, and 33.7 points in PA. Compared with these findings, the participants in this study scored higher in EE and DP and lower in PA, which may have resulted from the exceptional surge in COVID-19 infections in Korea. To illustrate, after the first confirmed patient was found in Korea in January 2020, the number of patients grew rapidly across the local community due to a large-scale group infection centering on a religious organization in late February [38,39]. This led to an increase in the number of screening sites and the establishment of strong measures against the spread of COVID-19. Therefore, the surge in patients may have influenced the burnout levels of HWs in this study.

In EE and DP, burnout was found to be worse for doctors and nurses than OHWs; it was worse for nurses than doctors in all three aspects of burnout. The burnout levels of nurses are worse than those from large-scale studies in other countries. To illustrate, a study conducted among 2014 Chinese nurses in February 2020 showed 23.44 in the mean score of EE, 6.77 in the mean score of DP, and 34.83 in the mean score of PA [13]. Likewise, another large-scale study carried out among 12,596 Chinese HWs in April 2020 showed 19.1 in the mean score of EE, 5.5 in the mean score of DP, and 29.0 in the mean score of PA [15]. In addition, a study carried out among HWs in Saudi Arabia [16] showed that nurses scored high in EE (24.70 points) and DP (8.37 points) compared to other job categories including doctors, who scored 22.85 points in EE and 7.36 points in DP. In PA, nurses scored low (34.22 points) compared to doctors (35.70 points).

### 4.3. Factors Influencing Emotional Exhaustion (EE)

The results of this study show that the factors affecting EE include sex, marital status, workload of treating suspected COVID-19 patients, fear of COVID-19 infection, anxiety, and depression. The level of EE is likely to increase under the following conditions: being female, single, having frequent contact with suspected COVID-19 patients, a strong fear of COVID-19 infection, and serious levels of anxiety and depression. In the hierarchical regression analysis, the psychological group demonstrated the greatest predictive power of EE. Similarly, the results of a study conducted among medical and administrative staff at a tertiary hospital in Italy during the COVID-19 pandemic [40] showed that the factors affecting the levels of EE, which was measured using the MBI-GS (General Survey), included sex, living condition, workplace, length of working experience, occupation, pre-existing psychological problems, COVID-19-related traumatic events, increased conflict among colleagues, additional task assignment, increased workload, and interpersonal avoidance. According to the study, females and people living alone had a higher level of EE compared to males and people living with family/other relatives. Comparatively, people working in administration, non-COVID wards, and frontline services caring for patients with COVID-19 had lower levels of EE than those working in an intensive care unit (ICU), frequently interacting with COVID-19 patients, and having a higher probability of infection. The results of the present study are consistent with another study conducted in Italy [37], which showed that being female, being in contact with COVID-19 patients, and fear of COVID-19 infection predicted increases in EE. Furthermore, the results of the present study are consistent with the findings of family division and trait anxiety in a study conducted among physicians and nurses in northern Italy [40]. A study conducted in Saudi Arabia [16] also showed that direct involvement in the management of COVID-19 patents increased the levels of EE.

Among the three job categories, there was a statistically significant difference in the EE scores. The results of the post hoc test show that the EE score for nurses was the highest. It is necessary to identify factors that increase nurses’ EE, such as anxiety, depression, fear of infection and death [13], and come up with measures to strengthen factors that decrease nurses’ EE, such as self-efficacy and resilience [13].

### 4.4. Factors Influencing Depersonalization (DP)

The factors that affected DP as a sign of burnout included nursing, working duration in the current department, satisfaction with work environment, anxiety, and depression. DP was worse for nurses than for doctors and OHWs. DP increased when the period of continuous service in the department was longer and when the satisfaction with the work environment was higher. Furthermore, DP levels increased as the levels of anxiety and depression increased. In the hierarchical regression analysis, the psychological group demonstrated the greatest predictive power of DP. A study conducted in Saudi Arabia [16] also found an association between DP and satisfaction with work environment and showed that DP levels are higher when working more than 8 h during the COVID-19 pandemic, when performing on-call duties, and when job duties are changed. In a study among health professionals in Italy [37], work hours were found to be one of the predictors of DP during the COVID-19 pandemic. In a study conducted in northwest Italy [41], DP during COVID-19 increased as the level of anxiety increased. The findings of this study are consistent with meta-analysis research [42], which reported that “greater experience through years worked” is one of the factors that decreases HWs’ risk of adverse psychological outcomes during virus outbreaks.

### 4.5. Factors Influencing Personal Accomplishment (PA)

The factors that affected PA included the workload of directly interacting with patients, socioeconomic status, and job stress. The levels of PA increased when interacting more frequently with patients and when socioeconomic status was higher. Meanwhile, PA decreased when job stress was high. In the hierarchical regression analysis, the psychological group demonstrated the greatest predictive power of PA. The results of this study are similar to the findings of a study conducted in Saudi Arabia [16], which suggested that PA levels during the COVID-19 pandemic are positively associated with direct involvement with the care of COVID-19 patients. The findings of this study are also supported by research conducted among emergency workers in Italy [43], which reported that emotional stress and cognitive stress decreased PA. According to a meta-analysis study [42], lower household income was included as one of the risk factors that increased HWs’ adverse psychological outcomes during virus outbreaks.

As it is ambiguous whether reduced PA is a result of or a manifestation of burnout [44], it is necessary to first consider the strategies that can be applied to both EE and DP. In this study, anxiety and depression were found to be predictors of both EE and DP. Therefore, first, it is necessary to discover the factors with the greatest impact on both anxiety and depression and, second, to establish and implement practical measures to relieve them. From an administrative standpoint, it is important to change the work environment so that hospital staff can feel that they are managing well autonomously without being overwhelmed by their jobs. This also helps them improve their work competency, job satisfaction, and mental health [45,46].

### 4.6. Comprehensive Strategies Considering the Three Components of Burnout

In addition to mitigating anxiety and depression, which are required for improving the levels of EE and DP, job stress is one of the factors that affects PA. Therefore, measures to lower psychological stress, simultaneously with anxiety and depression, should be considered. As preventive interventions, resilience, and social support were reported as mediator variables for psychological problems among HWs during the COVID-19 pandemic [47], it is essential to establish a policy that reflects these variables. To this end, it is imperative for individual employees and hospitals to collaborate and establish measures that can analyze and regulate the factors that cause anxiety, depression, and job stress during a pandemic by accurately diagnosing mental health conditions through counseling with professionals and operating a tailored counseling program.

It is also imperative to explore strategies to strengthen the resilience of hospital HWs to mitigate EE and DP, as well as to improve PA. Resilience can be defined as maintaining or recovering mental health during significant adversity, such as a potentially traumatic event, challenging life situation, major life change, or physical illness [48]. Furthermore, hardiness is a trait of resilience [49]. During the COVID-19 pandemic, resilience works as a protective factor and elicits a positive outcome by reducing burnout and stress [43] while improving performance, productivity, and satisfaction [50]. Therefore, by proactively introducing educational programs to improve and strengthen HWs’ hardiness and by offering these programs regularly, it will be possible for hospital staff to proactively counter and regulate their stress, stemming from work-related burden and interpersonal relationships, during a traumatic event such as COVID-19.

### 4.7. Response Policies at the National and Hospital Level

Currently, Korea is monitoring the mental health of frontline pandemic responders at the national level. The South Korean government launched the National Center for Disaster Trauma (NCDT) and began to operate the “COVID-19 Integrated Psychological Support Team” within a month after the COVID-19 pandemic began in January 2020. As the number of complaints about burnout related to COVID-19 increased among HWs and government officials, the NCDT and Korean Society of Traumatic Stress Studies published guidelines on psychosocial care for infectious disease management [51] and distributed them in March 2020. In addition to the guidelines, the Ministry of Health and Welfare and NCDT announced plans in May 2020 to provide burnout prevention education, counseling, and burnout management programs based on tailored consultation with individuals and organizations to prevent and monitor the job stress and burnout of COVID-19 responders [52].

In this study, satisfaction with the work environment was found to be a predictor of the levels of DP. Similarly, a safe and healthy working condition was found to mitigate DP in a study conducted among nurses in Malaysia [53], and a large-scale study conducted among nurses in China during COVID-19 also showed a significant statistical difference in DP depending on the level of work safety [13]. Moreover, a systematic review listed many attributes of the work environment that can mitigate DP as follows: pleasantness of tasks, value/meaning of work, emotional reward, and making a difference [54]. Thus, organizational management can mitigate the levels of DP by focusing on the following aspects: work hours, work environment, work patterns, work speed, work autonomy, education and training, communication within the organization, violence and discrimination, accidents and diseases, and work incentives [55].

According to this study’s findings, the workload of direct patient care was found to be a conflicting factor in improving EE and increasing PA. A high frequency of direct encounters with suspected COVID-19 patients increased EE, while a heavier workload of direct patient service improved PA. Thus, by expanding minimum contact systems and equipment, the burden of direct contact with suspected COVID-19 patients can be reduced. However, PA decreased because of a decrease in the workload of the direct patient service. Therefore, to maintain proper levels of PA, it is important to imitate and take advantage of direct patient services by providing contactless channels for HWs to sufficiently communicate with patients. For example, South Korea creatively developed and operated drive-through screening centers and walk-through screening centers [56,57]. The walk-through system is an efficient system that uses a booth to minimize the physical contact between the HWs and patients, protecting the medical staff with minimal protective equipment and shortening the testing time. Such a minimum contact system can help prevent passive treatment behavior due to a decrease in contact because of fear of infection. Nevertheless, it cannot be denied that reduced contact between HWs and patients can pose a danger to patients. Therefore, the degree of non-contact should be determined in line with the patient’s safety.

Burnout is a critical problem that adversely effects not only HWs, but also patients and the overall healthcare environment. When employee burnout is not properly monitored, it leads to intention to leave, reduced job performance, missed care, general health problems, mental health problems, and reduced job satisfaction; from the patients’ perspective, it can lead to poor quality of care, poor patient safety, adverse events, negative patient experience, medication errors, infections, and patient falls [58]. Therefore, preemptive burnout management is essential for hospital employees as well as for the effective treatment, care, and safety of patients. It is necessary to provide the opportunity for COVID-19 responders to debrief on their psychological experience and manage their mental health from the early stage of an outbreak of an infectious disease, since previous studies have shown that HWs display psychological trauma caused by epidemic outbreaks [59,60,61].

As such, the central and local governments’ countermeasures against the COVID-19 pandemic, which have continued from its early stages, are timely policies for reducing the burnout of COVID-19 responders and further preventing post-traumatic stress disorder (PTSD). These findings are in line with the WHO guidelines that highlight mental health and psychosocial support for HWs during the COVID-19 pandemic [62], as well as with the U.S. CDC guidelines [63] on how to cope with stress and build resilience for healthcare personnel and first responders.

### 4.8. Limitations and Suggestions for Follow-Up Studies

This study had the following limitations. First, this study limited its scope to the situation at one medical institution in one country during the COVID-19 outbreak, so caution should be exercised in generalizing the findings. Although this study’s findings cannot be applied to hospitals with different sizes and conditions, the research method used in this study can help identify the influencing factors of burnout components at different hospitals. Second, this study excluded some factors that can influence the burnout of hospital employees during a pandemic such as COVID-19. For example, other variables such as job demand, job control, value congruence, role conflict, decision latitude [58], or the presence of psychological comorbidities [33] should be considered in a follow-up study.

Based on the study findings, we suggest a stepwise approach that identifies the predictors of burnout components (EE, DP, and PA), selects the most vulnerable job category based on the identified predictors, and then manages the target job category, preferentially improving predictors that can exert a favorable influence on other job categories. We also suggest that follow-up studies should identify biomarkers and somatization in the workforce responding to infectious diseases by referring to the psychosomatic symptoms of burnout [64], biomarkers such as salivary cortisol, or biochemical parameters such as HbA1C [65].

## 5. Conclusions

During a pandemic of a novel infectious disease such as COVID-19, the government and hospital healthcare policy managers should consider the potential for burnout in HWs who first encounter patients and provide treatment. The results of this study show that, in the early stages of the response to COVID-19, the burnout (EE, DP, PA) levels of doctors and nurses at a general hospital were worse than that of other hospital HWs in EE and DP; the burnout levels of nurses were worse than those of doctors in all three aspects. The factors that affected EE related to COVID-19 were sex, marital status, fear of COVID-19 infection, anxiety, and depression; DP was affected by nursing, working duration in the current department, anxiety, and depression; and PA was affected by workload of directly interacting with patients, socioeconomic status, and job stress.

This study is significant for several reasons. First, this study referred to results from an exhaustive survey conducted among employees at one medical institution, who experienced the early stage of COVID-19; therefore, it was possible to identify and explain burnout mechanisms based on the characteristics of diverse job categories and work environments. Second, this study performed three different multiple regression analyses using EE, DP, and PA as the dependent variables, and identified significant factors for each component to enable the establishment of tailored policies according to the burnout component. The multi-perspective approach of this study can help establish macroscopic and comprehensive countermeasures at the institution level.

## Figures and Tables

**Table 1:** General characteristics, other variables, and differences in COVID-19-related burnout (*N* = 200).

Variable	Value	*n* (%)	EE Mean ± SD	*t*, *W*, or χ^2^ (*p*)	DP Mean ± SD	*t*, *W*, or χ^2^ (*p*)	PA Mean ± SD	*t*, *W*, or χ^2^ (*p*)
sex	male	47 (23.5)	19.30 ± 12.84	−4.80 (0.00)	8.60 ± 6.33	−2.85 (0.01)	29.02 ± 11.57	0.41 (0.68)
	female	153 (76.5)	28.69 ± 11.37		11.78 ± 6.81		28.30 ± 10.10	
age	<40	102 (51.0)	28.42 ± 11.08	2.28 (0.02)	11.68 ± 6.40	1.37 (0.17)	30.56 ± 8.53	2.92 (0.00)
	≥40	98 (49.0)	24.47 ± 13.33		10.36 ± 7.20		26.30 ± 11.77	
education level	high school	26 (13.0)	22.31 ± 10.98	7.48 (0.02)	11.23 ± 7.17	0.70 (0.71)	24.31 ± 9.78	7.11 (0.03)
	college	134 (67.0)	28.00 ± 12.84		11.23 ± 6.88		28.66 ± 10.26	
	graduate school	40 (20.0)	24.12 ± 10.74		10.22 ± 6.49		30.55 ± 10.94	
marital status	single	76 (38.0)	29.50 ± 12.20	2.74 (0.00)	12.14 ± 7.02	1.82 (0.07)	29.53 ± 8.26	1.21 (0.23)
	married	124 (62.0)	24.64 ± 12.14		10.35 ± 6.63		27.82 ± 11.55	
job category	doctor	48 (24.0)	25.67 ± 9.08	12.70 (0.00)	10.92 ± 5.33	4.52 (0.01)	32.25 ± 8.38	5.56 (0.01)
	nurse	83 (41.5)	31.30 ± 11.85		12.55 ± 7.09		27.86 ± 9.55	
	other health worker (OHW)	69 (34.5)	21.26 ± 12.82		9.28 ± 7.07		26.58 ± 12.09	
workload of directly interacting with patients	None	16 (8.0)	24.19 ± 12.08	9.06 (0.03)	12.12 ± 6.03	4.63 (0.20)	21.38 ± 9.90	15.12 (0.00)
	very few	37 (18.5)	21.57 ± 13.80		9.08 ± 7.57		24.70 ± 12.03	
	<1/4 of work hours	11 (5.5)	23.45 ± 10.97		9.45 ± 4.46		31.45 ± 7.02	
	≥1/4 of work hours	136 (68.0)	28.34 ± 11.74		11.56 ± 6.79		30.09 ± 9.71	
workload of treating suspected COVID-19 patients	none	40 (20.0)	20.70 ± 11.27	24.10 (0.00)	9.00 ± 6.35	10.22 (0.08)	26.45 ± 11.44	3.25 (0.36)
	very few	98 (49.0)	25.77 ± 12.10		10.61 ± 6.83		28.97 ± 10.60	
	<1/4 of work hours	33 (16.5)	30.09 ± 11.27		12.85 ± 6.66		30.67 ± 8.83	
	≥1/4 of work hours	29 (14.5)	32.79 ± 12.27		13.17 ± 6.85		27.07 ± 9.96	
socioeconomic status	high	26 (13.0)	24.38 ± 9.99	0.59 (0.56)	10.19 ± 5.39	0.18 (0.91)	34.88 ± 6.21	13.11 (0.00)
	middle	118 (59.0)	27.16 ± 12.04		11.19 ± 6.63		28.43 ± 9.45	
	low	56 (28.0)	26.04 ± 14.01		11.07 ± 7.83		25.57 ± 12.60	
satisfaction with work environment	very satisfied	7 (3.5)	18.57 ± 10.74	42.81 (0.00)	6.29 ± 5.41	31.15 (0.00)	23.86 ± 16.86	3.51 (0.32)
	satisfied	106 (53)	22.02 ± 10.83		8.86 ± 5.70		29.50 ± 9.97	
	dissatisfied	73 (36.5)	31.82 ± 10.40		13.73 ± 6.63		28.41 ± 9.66	
	very dissatisfied	14 (7.0)	36.43 ± 16.68		15.79 ± 8.43		23.29 ± 12.98	
current health condition	excellent	5 (2.5)	19.40 ± 9.32	41.34 (0.00)	6.80 ± 4.97	26.84 (0.00)	33.60 ± 9.66	2.35 (0.67)
	good	73 (36.5)	21.79 ± 10.89		9.55 ± 6.23		28.08 ± 11.00	
	average	84 (42.0)	26.46 ± 10.82		10.23 ± 5.77		29.01 ± 9.49	
	poor	35 (17.5)	35.51 ± 12.94		15.46 ± 7.62		27.51 ± 11.01	
	very poor	3 (1.5)	47.67 ± 5.69		25.00 ± 5.00		25.33 ± 19.66	
**Variable**			**Mean ± SD** **(** **range** **)**					
working duration in the current department (mth)			50.08 ± 66.45 (0–300)					
working duration in the current job (mth)			72.33 ± 75.83 (0–396)					
number of rotating shifts within a month (day)			7.50 ± 9.64 (0–24)					
job satisfaction			50.92 ± 13.75 (20–100)					
anxiety			7.49 ± 2.78 (0–21)					
depression			8.87 ± 3.34 (0–21)					
job stress			49.09 ± 10.60 (10.71–90.87)					
hospital resources for COVID-19			12.35 ± 3.22 (6–20)					
support from others			10.32 ± 2.27 (4–16)					
working overtime and compensation			6.38 ± 0.59 (5–8)					
EE score			26.48 ± 12.36 (0–54)					
DP score			11.03 ± 6.82 (0–30)					
PA score			28.47 ± 10.44 (0–48)					

**Table 2:** Multiple linear regression analysis for burnout (*N* = 200).

Burnout Component	Variable	*B*	*SE*	*β*	*t* (*p*)		CI	*F* (*p*)	*R* ^2^	*R*^2^ (adj)
EE	intercept	2.20	9.66		0.22	(0.82)		13.72 (<0.001)	0.56	0.52
	sex (female)	3.61	1.68	0.12	2.14	(0.03)	(0.29, 6.92)			
	age	−0.85	1.59	−0.03	−0.53	(0.60)	(−3.99, 2.30)			
	education level	0.33	1.21	0.02	0.28	(0.78)	(−2.05, 2.72)			
	marital status (married)	−3.07	1.45	−0.12	−2.12	(0.04)	(−5.92, −0.21)			
	nursing (yes)	−0.48	1.89	−0.02	−0.26	(0.80)	(−4.22, 3.25)			
	working duration in the current department	−0.02	0.01	−0.10	−1.63	(0.10)	(−0.04, 0.00)			
	number of shifts within a month	−0.08	0.08	−0.06	−1.00	(0.32)	(−0.24, 0.08)			
	workload of directly interacting with patients	−0.48	0.83	−0.04	−0.58	(0.56)	(−2.13, 1.16)			
	workload of treating suspected COVID-19 patients	2.05	0.76	0.16	2.70	(0.01)	(0.55, 3.54)			
	satisfaction with work environment	−1.59	1.26	−0.09	−1.26	(0.21)	(−4.07, 0.90)			
	current health condition	0.36	1.03	0.02	0.35	(0.73)	(−1.67, 2.39)			
	fear of COVID-19 infection	0.68	0.29	0.16	2.33	(0.02)	(0.10, 1.26)			
	job satisfaction	−0.07	0.06	−0.07	−1.09	(0.28)	(−0.19, 0.05)			
	anxiety	1.00	0.33	0.26	3.02	(<0.001)	(0.35, 1.65)			
	depression	0.96	0.29	0.26	3.37	(<0.001)	(0.40, 1.53)			
	job stress	0.13	0.09	0.11	1.49	(0.14)	(−0.04, 0.30)			
	hospital resources for COVID-19	0.16	0.27	0.04	0.58	(0.56)	(−0.38, 0.69)			
DP	intercept	4.10	5.60		0.73	(0.47)		9.92 (<0.001)	0.39	0.35
	sex (female)	0.68	1.25	0.04	0.63	(0.59)	(−1.78, 3.15)			
	nursing (yes)	−2.30	1.06	−0.17	−2.18	(0.03)	(−4.40, −0.20)			
	working duration in the current department	−0.09	0.01	−0.18	−2.84	(<0.001)	(−0.03, −0.07)			
	workload of treating suspected COVID-19 patients	0.66	0.45	0.09	1.47	(0.14)	(−0.23, 1.55)			
	satisfaction with work environment	−1.75	0.90	−0.17	−2.17	(0.05)	(−3.50, 0.01)			
	current health condition	0.20	0.74	0.02	0.31	(0.79)	(−1.26, 1.66)			
	fear of COVID-19 infection	0.13	0.16	0.04	0.62	(0.43)	(−0.19, 0.45)			
	job satisfaction	−0.02	0.04	−0.03	−0.42	(0.70)	(−0.10, 0.07)			
	anxiety	0.78	0.16	0.32	3.78	(<0.001)	(0.47, 1.10)			
	depression	0.32	0.17	0.16	1.76	(0.06)	(−0.02, 0.65)			
	job stress	0.04	0.05	0.06	0.68	(0.45)	(−0.06, 0.13)			
	hospital resources for COVID-19	0.02	0.16	0.01	0.13	(0.90)	(−0.30, 0.34)			
PA	intercept	42.64	10.58		4.03	(<0.001)		5.99 (<0.001)	0.18	0.15
	age	−1.54	1.62	−0.07	−0.98	(0.34)	(−4.74, 1.66)			
	education level	−0.78	2.07	−0.04	−0.51	(0.71)	(−4.87, 3.31)			
	doctor (yes)	0.70	2.62	0.03	0.32	(0.79)	(−4.46, 5.87)			
	workload of directly interacting with patients	1.94	0.89	0.19	2.44	(0.03)	(0.18, 3.70)			
	socioeconomic status	3.10	1.49	0.19	2.51	(0.04)	(0.15, 6.05)			
	job stress	−0.20	0.07	−0.21	−2.99	(<0.001)	(−0.35, −0.06)			
	working overtime and compensation	−2.22	1.54	−0.13	−1.67	(0.15)	(−5.25, 0.81)			

Note: Ordinal variables: education level, workload of directly interacting with patients, workload of treating suspected COVID-19 patients, satisfaction with work environment, current health condition, fear of COVID-19 infection, hospital resources for COVID-19. Integers 0 to *n* − 1 are assigned to the values of ordinal variables, where *n* is the number of values. Reference groups for dummy variables were sex (male), nursing (no), and doctor (no).

## Data Availability

The datasets generated and analyzed during the current study are not publicly available due to personally sensitive records but are available from the corresponding author upon reasonable request.
